# Outcomes of Intravenous Push versus Intermittent Infusion Administration of Cefepime in Critically Ill Patients

**DOI:** 10.3390/antibiotics12060996

**Published:** 2023-06-01

**Authors:** Susan E. Smith, Zachary Halbig, Nicholas R. Fox, Christopher M. Bland, Trisha N. Branan

**Affiliations:** 1 Department of Clinical and Administrative Pharmacy, University of Georgia College of Pharmacy, Athens, GA 30602, USA; 2 Department of Pharmacy, Piedmont Athens Regional, Athens, GA 30606, USA; 3 Athens Pulmonary, Piedmont Athens Regional, Athens, GA 30606, USA; 4 Department of Clinical and Administrative Pharmacy, University of Georgia College of Pharmacy, Savannah, GA 31405, USA

**Keywords:** cefepime, critical illness, drug administration routes, treatment failure, antibacterial agents, sepsis

## Abstract

The equivalence of intravenous push (IVP) and piggyback (IVPB) administration has not been evaluated in the critically ill population for most medications, but it is especially relevant for antibiotics, such as cefepime, that exhibit time-dependent bactericidal activity. A single center, retrospective, observational pre/post-protocol change study included critically ill adults who received cefepime as empiric therapy between August 2015 and 2021. The primary outcome was treatment failure, which was defined as a composite of escalation of antibiotic regimen or all-cause mortality. Secondary outcomes included adverse drug events, days of cefepime therapy, total days of antibiotic therapy, and ICU and hospital length of stay. Outcomes were compared using Chi-squared, Mann Whitney U, and binary logistic regression as appropriate. A total of 285 patients were included: 87 IVPB and 198 IVP. Treatment failure occurred in 18% (*n* = 16) of the IVPB group and 27% (*n* = 54) of the IVP group (*p* = 0.109). There were no significant differences in secondary outcomes. Longer duration of antibiotics (odds ratio [OR] 1.057, 95% confidence interval [CI] 1.013–1.103), SOFA score (OR 1.269, 95% CI 1.154–1.397) and IVP administration of cefepime (OR 2.370, 95% CI 1.143–4.914) were independently associated with treatment failure. Critically ill patients who received IVP cefepime were more likely to experience treatment failure in an adjusted analysis. The current practice of IVP cefepime should be reevaluated, as it may not provide similar clinical outcomes in the critically ill population.

## 1. Introduction

Cefepime is a fourth-generation cephalosporin commonly used in the treatment of sepsis and septic shock due to its broad spectrum of activity against both Gram-positive and Gram-negative bacterial pathogens, including *Pseudomonas aeruginosa* [1,2]. Cefepime is traditionally administered as an intravenous (IV) intermittent infusion (a.k.a., IV piggyback [IVPB]) over 30 min and has also been evaluated as a prolonged infusion when treating *P. aeruginosa* infections, especially those with higher minimum inhibitory concentrations (MICs) [3]. In late 2018, Hurricane Maria disrupted the production of small volume parenterals, leading to a national shortage [4,5,6]. As a way to alleviate this critical fluid shortage, some health systems transitioned standard administration of select IVPB medications to intravenous push (IVP) administration [7]. Some of these medications include cefazolin, ceftriaxone, cefepime, and meropenem.

IVP is generally defined as the administration of a final IV product injected over 5 min or less [5]. While the preparation parameters, stability, and administration instructions for IVP administration of cefepime have been established, the United States Food and Drug Administration (FDA) has not approved the use of cefepime administered as IVP, leading to limited data on IVP safety and efficacy [1]. The pharmacokinetic (PK) and pharmacodynamic (PD) effects of administering antibiotics IVP are largely unknown, particularly in the critically ill population, which is known to have significantly altered PK/PD, including increased volume of distribution and augmented renal clearance [8,9,10,11,12]. Additionally, IVP administration medications are given in a smaller, more concentrated volume over a shorter period of time. This technique could result in a higher risk of adverse events, such as phlebitis, infiltration, or neurotoxicity [2,4,13].

As infectious disease practice guidelines increasingly advocate for antimicrobial dosing strategies that optimize PK/PD parameters, it is essential to clinically evaluate safety and efficacy among various dosing strategies [2,14]. Cefepime is commonly used for empiric therapy in the ICU setting and also requires frequent dosing and demonstrates time-dependent bactericidal activity, which may make it particularly susceptible to PK alterations, leading to decreased efficacy. The purpose of this study is to examine the effects of cefepime administration strategies on antibiotic treatment failure and safety.

## 2. Results

**Figure 1:**
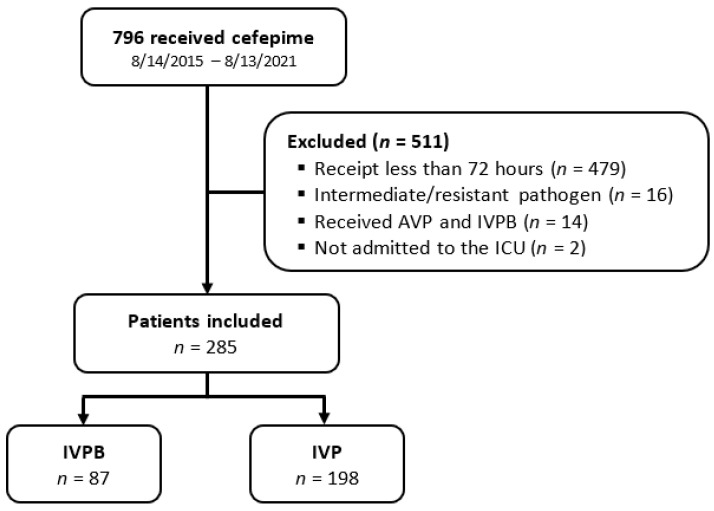
Patient enrollment. IVPB—intravenous piggyback; IVP—intravenous push; ICU—intensive care unit.

A total of 796 patients were screened for eligibility. Of those, 511 patients were excluded, primarily due to receipt of cefepime for less than 72 h. A total of 285 patients were included in the study. An amount of 87 patients were in the IVPB group, and 198 patients were in the IVP group (Figure 1). Patient characteristics were mostly similar between groups (Table 1). The IVPB group was older (73 vs. 67 years, *p* = 0.004) and had a lower creatinine clearance at the time of cefepime initiation (37 vs. 49 mL/min, *p* = 0.049). Sepsis was more common in the IVPB group (83% vs. 69%, *p* = 0.044), but septic shock was more common in the IVP group (17% vs. 31%, *p* = 0.044). Pneumonia was the most commonly identified source of infection in both groups, accounting for over 50% of infections. Pathogens were isolated in 151 (53%) of patients, with the most commonly isolated pathogen being *Pseudomonas aeruginosa* (*n* = 47).

Treatment failure occurred in 18% (*n* = 16) of the IVPB group and 27% (*n* = 54) of the IVP group (*p* = 0.109), with escalation of therapy occurring in 2% (*n* = 2) versus 9% (*n* = 18) (*p* = 0.093), and all-cause mortality occurring in 18% (*n* = 16) versus 22% (*n* = 44) (*p* = 0.339) (Table 2). Nine patients experienced both escalation of antibiotic therapy and mortality: one in the IVPB group and eight in the IVP group. A post hoc power calculation, based on the number of patients included and the observed rate of treatment failure, indicated that there was 36.4% power to detect a difference in treatment failure between the two groups with a significance level of 0.05.

The average daily dose of cefepime was higher in the IVP group overall (3.3 vs. 3.8 g/day, *p* < 0.001) and for the subgroups with CrCl > 60 mL/min and with CrCl 30–60 mL/min, but not in the subgroup with CrCl < 30 mL/min. There was no significant difference in total days of cefepime (six vs. six days, *p* = 0.314) or antibiotic therapy (nine vs. ten days, *p* = 0.194) or in the intensive care unit (ICU) (six vs. seven days, *p* = 0.06) or hospital length of stay (eleven vs. thirteen days, *p* = 0.148). There was also no significant difference in drug-induced ADR (*p* = 0.915) (Table 2). Drug-induced ADR occurred in one patient in the IVPB group who had slightly reduced kidney function (CrCl 54 mL/min), received an average of 3.33 g of cefepime per day, and experienced altered mental status. Antibiotic therapy was considered complete, and antibiotics were discontinued. Of note, the patient was receiving concomitant levofloxacin. Drug-induced ADRs occurred in two patients in the IVP group who had an average CrCl of 76.5 mL/min and received an average daily dose of 3.65 g of cefepime per day. One of these patients experienced altered mental status, and the other patient experienced a petechial rash that developed within 3 h of administration. In both cases, the antibiotic was changed to meropenem.

When controlling for potentially confounding variables through a pre-specified binary logistic regression, longer duration of antibiotics (OR 1.057, 95% CI 1.013–1.103), higher Sequential Organ Failure Assessment (SOFA) score (OR 1.274, 95% CI 1.157–1.404), and IVP administration of cefepime were independently associated with treatment failure (OR 2.4, 95% CI 1.149–5.017) (Table 3).

In the subgroup of 47 patients with *Pseudomonas aeruginosa*, 14 received cefepime via the IVPB route, and 33 received it via IVP. The incidence of treatment failure was 21% (*n* = 3) in the IVPB group and 48% (*n* = 16) in the IVP group (*n* = 0.084). Escalation of antibiotic therapy occurred in 0 patients in the IVPB group and in 15% (*n* = 5) of the IVP group (*p* = 0.123), and hospital mortality occurred in 21% (*n* = 3) and 39% (*n* = 13) of the two groups (*p* = 0.745). Demographic variables were similar between the two groups. The average daily dose was also similar between groups (IVPB: 3.6 [IQR 1.9–4.9] vs. IVP: 4.0 [3.0–5.3] grams, *p* = 0.305). In multivariate regression, SOFA score (OR 1.640, 95% CI 1.205–2.233) and IVP route of administration (OR 11.860, 95% CI 1.158–121.495) were independent risk factors for treatment failure.

## 3. Discussion

In this retrospective pre/post-protocol change study, IVP administration of cefepime was associated with 2.4 times increased risk of treatment failure compared to IVPB administration after adjusting for patient demographics, severity of illness, and characteristics of the infection. The individual components of the composite outcome (i.e., all-cause mortality and escalation of antibiotic therapy) were similar between the two groups in unadjusted analyses. The rate of ADEs was also similar with IVP and IVPB administration.

Standard infusions of most intravenous beta-lactams are between 30 and 60 min, depending on the specific medication. This includes once daily agents, such as ertapenem and ceftriaxone, which are often used in the outpatient setting due to their convenient dosing schedules. Due to persistence or even increased incidence of resistant Gram-negative organisms, such as multidrug-resistant *P. aeruginosa*, alternative dosing strategies for beta-lactams have been evaluated and used in clinical practice over the last 10 to 15 years [15]. Increasing antibiotic resistance is further exacerbated by a decrease in antimicrobial research and development, leading to few novel mechanism-of-action compounds being approved by regulatory agencies [16]. Consideration for beta-lactam therapeutic drug monitoring in the critically ill is also being considered due to the PK complexity within an individual patient [17]. Due to most beta-lactams possessing short half-lives and exhibiting time-dependent killing, most augmentation PK/PD dosing strategies have involved either extending the infusion time to 3 or 4 h or converting to a continuous infusion strategy [18]. Several newer beta-lactams, including cefiderocol, ceftazidime/avibactam, and meropenem/vaborbactam, have been FDA-approved as extended infusions over two to three hours, depending on the agent. Many inpatient facilities have adopted the extended infusion strategy for piperacillin-tazobactam, either for every patient or selected patients (e.g., ICU patients), while cefepime has been infused as an extended infusion, typically on an as-needed basis, often as a 3-h infusion for higher MIC pathogens [3,18]. Continuous infusion strategies are difficult to implement in the inpatient setting due to drug incompatibility logistics of occupying an intravenous line continuously, which relegates their use primarily for outpatient parenteral antimicrobial therapy.

Due to climbing MICs for many Enterobacterales species, regulatory agencies, such as the Clinical Laboratory Standards Institute (CLSI) and the FDA, have modified susceptibility breakpoint recommendations in recent years, incorporating a “susceptible dose-dependent” category [19]. These are currently recommended for a number of agents including piperacillin/tazobactam and cefepime for MICs of 16/4 mcg/mL and 4–8 mcg/mL, respectively. For isolates with these MICs, either three- or four-hour infusions are recommended for piperacillin/tazobactam, while a dose of 2 g IV q8h is recommended for cefepime, using the standard infusion time of 30 min. The overall trends in dosing regimens are for extending infusion times to maximize drug concentration target attainment after the first IVP dose, which is often given to critically ill patients.

Despite the trend toward longer infusion times, the IVP administration strategy offers several potential clinical and operational advantages [20]. It eliminates the need for small volume parenterals, which have intermittently been on drug shortage lists, dating back to 2007 [4]. IVP administration may also decrease the time to administration of first dose antibiotics, which is known to be of particular importance in patients with septic shock [21]. Additionally, IVP limits the volume of fluid administered. This objective is often recognized in fluid-restricted patients, such as those with acute heart failure exacerbation, resulting in volume overload or with acute renal failure, but it is important in all critically ill patients, in which IV medications comprise over 40% of total fluid intake in the first three days of ICU admission, often in the form of “hidden fluids”, where the fluid volume is not specifically prescribed [22]. These potential benefits must be weighed against possible risks. It is important to keep in mind that IVP administration is not currently FDA-approved and is considered an off-label use. Optimal timing of the second dose, even if transitioning to IVPB administration, is currently unstudied.

Outcome and adverse event data with IVP administration of cefepime are lacking, with most of the published data focusing on time to antibiotic administration, rather than clinical outcomes. In a pre/post protocol change study examining first-dose IVP cephalosporins in the emergency department, McLaughlin et al. found a 14 min decrease in time to cefepime administration (*p* < 0.007), reduced supply costs, and increased nursing satisfaction with IVP administration compared to IVPB [23]. Tran et al. evaluated over 1000 patients receiving combination of cefepime and vancomycin in the emergency department and found that giving cefepime via IVP resulted in a statistically significant decrease in time to vancomycin administration, with a median time from administration of cefepime to vancomycin of 63.5 min in the IVPB group, compared to 2 min in the IVP group (*p* < 0.001) [24]. These trials solely focused on the first dose of cefepime or concomitant antibiotics in an emergency department setting. Wiskirchen et al. compared a rapid 5-min infusion versus the standard 30-min infusion of ertapenem over a three-day period. The authors concluded that the IVP and IVPB administration regimens were bioequivalent and pharmacodynamically equivalent and were both well tolerated; however, it should be noted that this prospective, randomized, crossover pharmacokinetic study was conducted in 12 healthy volunteers and not in patients with an active infection, much less a critically ill population [25]. To our knowledge, no previously published studies have evaluated patient outcomes, such as escalation of antibiotic therapy or mortality with IVP administration of cefepime.

Several recent trends highlight the need for clinical evaluation of cefepime administration strategies. The creation of the susceptible dose-dependent category by CLSI effectively lowered the cefepime susceptibility breakpoint for Enterobacterales from ≤8 mcg/mL to ≤2 mcg/mL, making drug concentration target attainment more challenging [19]. In addition, some infectious disease guidelines advocate for dosing strategies that optimize PK parameters [2,14]. Based on these developments, it is essential to evaluate various cefepime dosing regimens to ensure improved logistical administration strategies are not compromising patient outcomes [2,10]. Cefepime exhibits time-dependent bactericidal effects, which may be attenuated utilizing the IVP route due to a shorter time of free drug, exceeding the MIC. These potential effects may be compounded in the critically ill population, which is known to exhibit an increased volume of distribution, hypoalbuminemia, augmented renal clearance or rapidly changing renal function, increased MICs to many pathogens, and/or multi-organ failure [8,9,10,11]. Recently, Barreto et al. demonstrated that 70% of critically ill patients treated with cefepime had inadequate cefepime exposure during the first 24 h of therapy [26].

To our knowledge, only two previous trials have studied PK differences with cefepime via the IVP route. Butterfield-Cowper et al. performed Monte Carlo simulations comparing IVP and IVPB administration routes. Exposure profiles were estimated using two-compartment population PK models of 35 patients who received cefepime for a documented or presumed infection. The authors found no difference in probability of target attainment against the range of MICs tested when cefepime administration was simulated over 5 min compared to 30 min [27]. They discussed that reducing the infusion time by 25 min should shorten the time to maximum concentration, but have minimal effect on concentration time profile and time above MIC. While this study suggests similar PK profiles with IVP and IVPB administration, it is limited by its small sample size and lack of clinical endpoints, such as efficacy and adverse effects. Liu et al. similarly performed Monte Carlo simulations to compare cefepime IVP administration over 2 min to IVPB administration over 30 min [28]. The two-compartment population PK model was covariate-adjusted for weight and creatinine clearance and was developed from 70 patients with 604 cefepime concentrations. In contrast to Butterfield-Cowper, Liu and colleagues found a 2.3% lower probability of target attainment with IVP administration for MICs of 0.25–0.5 mg/L and a 5.4% lower probability of target attainment for MICs of 1–4 mg/L. There was no difference in probability of target attainment with MICs of 8 mg/L or higher; however, neither administration technique reached the PK goal of probability of target attainment greater than 70%. Across renal functions defined by creatinine clearance of 60, 100, and 140 mL/min, probability of target attainment was lower with higher creatinine clearance. The authors concluded that unintended clinical consequences could occur as a result of IVP administration and that clinicians should exercise caution when using this administration strategy. To our knowledge, no patient-centered studies have confirmed either of these simulation-based PK findings.

Several patient-centered trials have examined outcomes with standard administration of cefepime. In a meta-analysis of 57 trials that compared cefepime to a different β-lactam antibiotic, cefepime was associated with a higher all-cause mortality at 30 days compared to other beta lactams in the treatment of sepsis (RR 1.26, 95% CI 1.08–1.49) [29]. The authors were unable to identify a specific cause for the increase in mortality or identify a specific patient population at risk; hypotheses include the potential for higher cefepime MICs that are more difficult to treat and the administration technique, suggesting that continuous administration could have been superior to IVPB. Bauer et al. compared a standard 30-minute cefepime infusion to an extended 4-h infusion in the treatment of *Pseudomonas aeruginosa* pneumonia and/or bacteremia. Overall, mortality was 20% with the standard infusion compared to 3% with the extended infusion (*p* = 0.03) [3]. In a propensity score-matched cohort study, Wang et al. observed a 2.87-time higher risk of death at 14 days compared to carbapenems when treating invasive extended spectrum beta-lactamase (ESBL) bacteremia [30]. Both of these trials demonstrate the importance of applying PK/PD parameters to optimize efficacy, especially when treating more severe infections, such as *P. aeruginosa* and ESBL-producing organisms.

To our knowledge, this is the first study to compare outcomes and adverse effects between the IVP and IVPB administration of cefepime in critically ill patients. Although no difference was seen in primary or secondary outcomes initially, after completing the pre-specified binary logistic regression accounting for potential confounders, a significant difference in treatment failure with the IVP group was observed. The difference in treatment could have resulted from IVP administration decreasing the time above the MIC and, therefore, decreasing bactericidal activity or from altered pharmacokinetics that are known to be present in critically ill patients. These differences could be exacerbated when treating more complex infections, such as ESBLs or *Pseudomonas aeruginosa*, in which extended infusions have been shown to decrease mortality [29,30].

IVP administration poses the potential for adverse drug events due to the infusion of a more concentrated drug product over a shorter duration of time. Evaluation of the safety of IVP administration is limited in previous reports. In the present study, there was no significant difference in the number of adverse drug events between the groups, which aligns with the previous literature from Garrelts et al. that found no clinically significant differences in rates of adverse events, injection site reaction, vital signs, or laboratory parameters with administration of cefepime via IVP in healthy subjects [31]. It should be noted, however, that, in this study “IV bolus” infusion over 3 min involved delivery of 50 mL infusion volume, which is different from the more concentrated volume of IVP delivery administered in the present study. A retrospective review of 1000 patients who received IVP aztreonam, ceftriaxone, cefepime, or meropenem was published, evaluating safety [32]. Patients were administered cefepime IVP over 5 min with 10 mL of diluent per 1 g of medication. While overall beta lactams given IVP were well tolerated, over half of the adverse effects observed were with cefepime, including four cases of neurotoxicity manifested by altered mental status and myoclonic jerking. The number of doses received prior to the ADE ranged from three to twenty-three across the four patients. According to the Naranjo criteria, three of these cases were “possible”, and one was “probable”, with three of four patients having appropriate renal dose adjustments. Each case had the IVP cefepime administered via peripheral venous line and required discontinuation of medication and a switch to an alternative agent. Additionally, Foong et al. recently reported a cluster of patients with cefepime-induced neutropenia during outpatient administration of cefepime [33]. Neutropenia was more common in patients who received a prolonged course of cefepime of two weeks or more and who received cefepime by the IVP administration route. Patients that experienced cefepime-induced neutropenia were three times more likely to receive IVP cefepime compared to patients that did not experience neutropenia, with a relative risk of 9.28 (95% CI 2.00–43.06). An association between IVP cefepime and adverse effects was not observed in the present study, although evaluation of adverse drug events was limited in a retrospective, observational study that relies on documentation of adverse drug events in the medical record. Evaluation of adverse drug events that are rare, such as cefepime-induced neutropenia or neurotoxicity, also requires a much larger sample size than was available in this report [34].

A post hoc subgroup analysis was conducted to further examine patients with infections caused by *Pseudomonas aeruginosa* due to the prior literature, highlighting the importance of PK/PD in optimizing antibiotic efficacy in this population. Treatment failure was numerically higher for patients with *P. aeruginosa* than the overall cohort (40% vs. 25%) and was twice as high in patients with *P. aeruginosa* who received IVP compared to IVPB administration. Escalation of antibiotic therapy and mortality were also numerically higher in the IVP group. The adjusted analysis, controlling for severity of illness, age, gender, source of infection, and duration of antibiotic therapy, was even more striking, demonstrating that patients infected with *P. aeruginosa* who received IVP administration of cefepime were over 11 times more likely to experience treatment failure than patients receiving IVPB administration. While interpretation is limited by the small sample size (*n* = 47) and post hoc analysis, these findings are concerning. It would be cautious to avoid IVP administration of cefepime in patients with confirmed *P. aeruginosa* infection in the absence of larger scale studies confirming the efficacy of this practice.

Limitations of this study include the single center design, retrospective nature, and reliance on documentation in the electronic health record, all of which could lead to potential bias. Documentation of ADEs, in particular, may not be performed in a consistent manner and could be under-reported in this study. Prospective reporting of ADEs would be more accurate, but it was not feasible. Delirium, as reported by the confusion assessment method for the ICU (CAM-ICU), would have been a good surrogate for neurotoxicity, but it was not available, as this assessment is not documented reliably in the EHR. Although mortality between the two groups was similar, there was a higher incidence of sepsis in the IVPB group and of septic shock in the IVP group, which could have skewed the results. Additionally, an a priori sample size calculation could not be performed due to the lack of previous studies in this area, and a post hoc power calculation demonstrated only 36.5% power to detect a difference in the primary outcome, indicating that a type two error could have occurred. Composite outcomes have been attributed to an exaggeration in the perceived benefit of study interventions [35]. The composite outcome of treatment failure evaluated in this study, however, was defined to be consistent with that used in the previously published literature, evaluating clinical outcomes of cephalosporin therapy in critically ill patients [36]. There was also a significant difference in the number of patients included in the two groups, with about twice as many patients in the IVP group. Patients were enrolled during the same time duration (IVPB: 14 August 2015 through 13 August 2018; IVP: 14 August 2018 through 13 August 2021), suggesting that use of cefepime increased during recent years. Hypotheses for this difference include prescriber habits, updating of order sets to include cefepime as first line therapy during the three-year IVP time frame, increased incidence of piperacillin/tazobactam resistance within Enterobacterales, including *P. aeruginosa*, and newer evidence, suggesting an increased incidence of AKI with concomitant vancomycin and piperacillin/tazobactam. The study groups were defined based on periods of time in which each of the cefepime administration methods were used at the study site, with a change from IVPB to IVP administration for all patients occurring on 14 August 2018. As a result, there are no instances of IVPB and IVP administration of cefepime within the same time period. It is possible that other factors occurring over this time period, such as the advancement of medical care, may influence patient outcomes. One would expect, however, to see a lower incidence of treatment failure in the more recent years, which is the opposite of what was observed. Finally, a lower average daily dose of cefepime was observed in the IVPB group compared to the IVP group. While the reason for this lower dosing is unclear, one might expect the group that received higher doses to experience a lower incidence of treatment failure; this was not the case. Despite these limitations, this is the first study to our knowledge that examined clinical outcomes associated with IVP administration of cefepime.

## 4. Materials and Methods

### 4.1. Study Design, Site, and Population

This is a single center, retrospective, observational pre/post-protocol change study of patients who received cefepime for empiric therapy by the IVPB or the IVP route. The study was conducted at Piedmont Athens Regional, a 360-bed community teaching hospital in Athens, GA, USA. The standard empiric dose of cefepime was 2 g IV every 8 h for critically ill patients, which was adjusted per the manufacturer’s recommendations when calculated creatinine clearance was less than 60 mL/min. Although this was the standard dose used, other doses could be ordered by providers, and patients receiving all dosing regimens were included in this analysis. IVPB doses were prepared in a total volume of 100 mL and were administered by infusion pump over 30 min. IVP doses were reconstituted with 10 mL of sterile water for injection and were administered by the patient’s bedside nurse as a slow intravenous push over 5 min. The study protocol was reviewed by the hospital’s Institutional Review Board (IRB) and was determined to be exempt from IRB oversight. Patients were identified by the pharmacy dispensing logs of cefepime between 14 August 2015 and 13 August 2021. The institution changed its standard practice for cefepime infusion on 14 August 2018. Prior to this date, cefepime was administered by IVPB and, after this date, cefepime was administered by IVP. Therefore, all patients observed between 2015 and 13 August 2018 received IVPB cefepime, and all patients observed between 14 August 2018 and 2021 received IVP cefepime. Patients were included if they were at least 18 years of age and admitted to the ICU. Patients infected with a pathogen intermediate or resistant to cefepime (including susceptible-dose-dependent), pregnant patients, those that received cefepime for less than 72 h duration (including administration in the Emergency Department), and those that received cefepime through both IVP and IVPB routes, were excluded.

### 4.2. Data Collection

Baseline characteristics collected at the time of cefepime initiation via chart review of the electronic medical record included: age, race/ethnicity, gender, height and weight, history of penicillin and/or cephalosporin allergy, renal function (SCr and calculated CrCl via Cockcroft-Gault), and SOFA score. Clinical datapoints included: source of infection, positive rapid diagnostic results, pathogen if one was isolated on culture, and presence of sepsis and/or septic shock, as described in provider notes. Source of infection was categorized as follows: pneumonia (including pleural infections), intra-abdominal, urinary tract infection, skin and soft tissue infection (wound, abscess, cellulitis, open fracture), severe infections (bacteremia, central nervous system infection, osteomyelitis, endocarditis), multiple sites of infection, and unclear source of infection. Unclear source of infection was defined as no focal source of infections located via chart review or in provider notes. Average daily cefepime dose, duration of therapy, and adverse events were also collected. Notes documented in the medical record on the day of cefepime discontinuation and on the day of transfer/discharge from the ICU were reviewed for adverse drug events. While mention of any adverse events was considered, specific events being screened for included neurotoxicity, infusion reaction, neutropenia, and allergic reaction. Other antibiotic data, such as escalation of therapy, change in antibiotic due to tolerability or adverse events, and the total duration of all antibiotic therapy used, were also collected.

### 4.3. Outcomes

The primary outcome was treatment failure, defined as a composite of inpatient mortality and/or switching from cefepime to a broader Gram-negative antibiotic (i.e., carbapenem) due to clinical worsening, as documented in the electronic medical record (EMR). Secondary outcomes included adverse drug events (as documented in the EMR), days of cefepime therapy, total days of antibiotic therapy, ICU and hospital length of stay, and ICU and hospital mortality.

### 4.4. Statistical Analysis

Statistical analyses were performed using IBM^®^ SPSS Statistics, Version 28. All outcomes were compared between patients who received cefepime through the IVPB and IVP administration routes. Categorical variables, including the primary outcome, were compared using the Chi-squared test and are reported as number and percentage. Continuous variables were evaluated for their distribution and were compared using the independent two-sample *t*-test and reported as mean and standard deviation if normally distributed, or they were compared using the Mann-Whitney U test and reported as median and interquartile range if non-parametrically distributed. The sample size could not be predetermined, as this was a retrospective study, and the number of patients included was dependent on the timeframe of available data. Therefore, a post hoc power calculation was performed based on the available sample size and the observed rate of treatment failure. A multivariate binary logistic regression model was applied to the primary outcome to identify variables associated with treatment failure. The dependent variable in the model was treatment failure. Independent variables included in the model were agreed upon by consensus of the investigators a priori and included age, gender, source of infection, duration of antibiotic therapy, SOFA score, and route of administration (IVP vs. IVPB). Both the unadjusted, univariate analysis and adjusted, multivariate analysis are reported. A post hoc subgroup analysis was conducted to further examine patients with a culture positive for Pseudomonas aeruginosa. All statistical tests were repeated in this subgroup. For all analyses, an alpha less than 0.05 was considered significant.

## 5. Conclusions

Critically ill patients had a similar rate of treatment failure with IVPB and IVP administration of cefepime. Treatment failure was more likely with IVP administration of cefepime in an adjusted analysis. Current practice of IVP administration of cefepime should be further evaluated in the critically ill population. Future research will evaluate the suspected differences in PK parameters between these two administration routes and will compare these administration routes in non-critically ill patients.

## Figures and Tables

**Table 1:** Baseline patient characteristics.

Variable	IV Piggyback (*n* = 87)	IV Push (*n* = 198)	*p*-Value
Age	73 (63–81)	67 (58–76)	0.004 *
Height (cm)	172.4 (162.0–182.2)	170.2 (165.1–180.3)	0.795
Weight (kg)	87 (67–109)	82.7 (70.1–100)	0.709
Male Gender	46 (53)	116 (59)	0.370
Race/Ethnicity			
Caucasian	71 (82)	142 (72)	0.532
African-American	16 (18)	52 (26)	
Hispanic	0	1 (0.5)	
Asian	0	1 (0.5)	
Unknown	0	1 (0.5)	
SCr (mg/dL)	1.42 (0.92–2.77)	1.13 (0.85–1.83)	0.087
CrCl (mL/min)	37 (16–64)	49 (27.1–84.5)	0.049 *
SOFA Score	6 (3–8)	5 (2.75–7)	0.184
Source of Infection			
Pneumonia	48 (55)	101 (51)	0.267
Intraabdominal	1 (1)	13 (7)	
Urinary tract	7 (8)	9 (5)	
Severe ^a^	7 (8)	20 (10)	
Skin and soft tissue	4 (5)	15 (8)	
Multiple sources	20 (23)	40 (20)	
Sepsis	72 (83)	137 (69)	0.044 *
Septic Shock	15 (17)	61 (31)	0.044 *
Isolated Pathogen(s)			
*Pseudomonas aeruginosa*	14 (16)	33 (17)	0.904
Methicillin-resistant *Staphylococcus* spp.	4 (5)	9 (5)	0.984
Methicillin-susceptible *Staphylococcus* spp.	3 (3)	10 (5)	0.550
*Streptococcus* spp.	4 (5)	8 (4)	0.829
*Klebsiella* spp.	3 (3)	9 (5)	0.671
*Escherichia coli*	2 (2)	6 (3)	0.731
*Proteus* spp.	3 (3)	5 (3)	0.664
*Enterobacter* spp.	5 (6)	2 (1)	0.017 *
*Serratia* spp.	4 (5)	2 (1)	0.052
*Bacteroides*	2 (2)	1 (1)	0.172
*Citrobacter* spp.	2 (2)	1 (1)	0.172
*Haemophilus* spp.	1 (1)	1 (1)	0.548
Other bacteria ^b^	5 (6)	11 (6)	0.948
Polymicrobial	9 (10)	17 (9)	0.635

All data are presented as no. (%) or median (interquartile range). SCr—serum creatinine; CrCl—creatinine clearance; SOFA—sequential organ failure assessment. ^a^ Severe infections are defined as bacteremia, central nervous system infection, osteomyelitis, or endocarditis. ^b^ Other bacteria include *Moraxella*, *Burkholderia*, *Morganella*, *Acinetobacter*, *Stenotrophomonas*, and *Elizabethkingia* spp. * indicates *p* < 0.05.

**Table 2:** Primary and secondary outcomes.

Variable	IV Piggyback (*n* = 87)	IV Push (*n* = 198)	*p*-Value
Treatment failure	16 (18)	54 (27)	0.109
Escalation of therapy	2 (2)	18 (9)	0.093
All-cause mortality	16 (18)	44 (22)	0.339
Adverse drug event	1 (1)	2 (1)	0.915
Therapy change due to ADE	1 (1)	2 (1)	0.915
Average cefepime daily dose, g	3.33 (2–4)	3.785 (2.65–5)	<0.001 *
CrCl > 60 mL/min			
Number	28 (32)	76 (38)	
g/day	3.84 (3.50–5.00)	4.82 (3.68–6.00)	0.019 *
CrCl 30–60 mL/min, g/day			
Number	26 (30)	65 (33)	
g/day	3.46 (2.00–4.05)	4.00 (3.29–4.84)	0.013 *
CrCl < 30 mL/min, g/day			
Number	33 (38)	57 (29)	
g/day	2.00 (1.24–2.74)	2.00 (1.14–3.46)	0.716
Duration of cefepime, days	6 (4–8)	6 (5–8)	0.314
Duration of antibiotics, days	9 (6–12)	10 (7–14)	0.194
ICU LOS, days	6 (2–10)	7 (4–14)	0.060
Hospital LOS, days	11 (8–22)	13 (9–22)	0.148

All data are presented as no. (%) or median (interquartile range). ADE—adverse drug event; ICU—intensive care unit; LOS—length of stay. * indicates *p* < 0.05

**Table 3:** Logistic regression of factors associated with treatment failure.

	Univariate Analysis		Multivariate Analysis	
Variable	Odds Ratio (95% CI)	*p*-Value	Odds Ratio (95% CI)	*p*-Value
Age	1.005 (0.985–1.025)	0.648	1.013 (0.989–1.037)	0.287
Female gender	0.910 (0.527–1.573)	0.737	1.248 (0.673–2.312)	0.482
Source of Infection				
Pneumonia	Reference	n/a	Reference	n/a
Intraabdominal	0.190 (0.024–1.495)	0.114	0.082 (0.009–0.770)	0.029 *
UTI	0.164 (0.021–1.283)	0.085	0.260 (0.031–2.156)	0.212
Skin and Soft Tissue	0.657 (0.206–2.094)	0.478	0.634 (0.176–2.285)	0.486
Multiple Sources	0.682 (0.336–1.386)	0.290	0.435 (0.192–0.982)	0.045 *
Severe Infection	1.038 (0.422–2.550)	0.935	0.704 (0.261–1.895)	0.487
Duration of antibiotic therapy	1.074 (1.033–1.116)	<0.001 *	1.057 (1.013–1.103)	0.011 *
SOFA score	1.246 (1.144–1.357)	<0.001 *	1.269 (1.154–1.397)	<0.001 *
IVP administration (compared to IVPB)	1.664 (0.890–3.112)	0.111	2.370 (1.143–4.914)	0.020 *

CI—confidence interval; SOFA—sequential organ failure assessment; IVP—IV push; IVPB—IV piggyback. * indicates *p* < 0.05.

## Data Availability

The data presented in this study are available upon request from the corresponding author. The data are not publicly available due to patient privacy.
